# Highly divergent CRESS DNA and picorna-like viruses associated with bleached thalli of the green seaweed *Ulva*

**DOI:** 10.1128/spectrum.00255-23

**Published:** 2023-09-19

**Authors:** Luna M. van der Loos, Lander De Coninck, Roland Zell, Sebastian Lequime, Anne Willems, Olivier De Clerck, Jelle Matthijnssens

**Affiliations:** 1 Phycology Research Group, Department of Biology, Ghent University, Ghent, Belgium; 2 Laboratory of Microbiology, Department Biochemistry and Microbiology, Ghent University, Ghent, Belgium; 3 Laboratory of Clinical and Epidemiological Virology, Laboratory of Viral Metagenomics, Department of Microbiology, Immunology and Transplantation, Rega Institute, KU Leuven, Leuven, Belgium; 4 Section of Experimental Virology, Institute for Medical Microbiology, Jena University Hospital, Friedrich Schiller University, Jena, Germany; 5 Cluster of Microbial Ecology, Groningen Institute for Evolutionary Life Sciences, University of Groningen, Groningen, The Netherlands; University of Mississippi, University, Mississippi, USA

**Keywords:** seaweed, *Ulva*, chlorophyta, RNA viruses, DNA viruses

## Abstract

Marine macroalgae (seaweeds) are important primary producers and foundation species in coastal ecosystems around the world. Seaweeds currently contribute to an estimated 51% of the global mariculture production, with a long-term growth rate of 6% per year, and an estimated market value of more than US$11.3 billion. Viral infections could have a substantial impact on the ecology and aquaculture of seaweeds, but surprisingly little is known about virus diversity in macroalgal hosts. Using metagenomic sequencing, we characterized viral communities associated with healthy and bleached specimens of the commercially important green seaweed *Ulva*. We identified 20 putative new and divergent viruses, of which the majority belonged to the Circular Rep-Encoding Single-Stranded (CRESS) DNA viruses [single-stranded (ss)DNA genomes], *Durnavirales* [double-stranded (ds)RNA], and *Picornavirales* (ssRNA). Other newly identified RNA viruses were related to the *Ghabrivirales*, the *Mitoviridae*, and the *Tombusviridae*. Bleached *Ulva* samples contained particularly high viral read numbers. While reads matching assembled CRESS DNA viruses and picorna-like viruses were nearly absent from the healthy *Ulva* samples (confirmed by qPCR), they were very abundant in the bleached specimens. Therefore, bleaching in *Ulva* could be caused by one or a combination of the identified viruses but may also be the result of another causative agent or abiotic stress, with the viruses simply proliferating in already unhealthy seaweed tissue. This study highlights how little we know about the diversity and ecology of seaweed viruses, especially in relation to the health and diseases of the algal host, and emphasizes the need to better characterize the algal virosphere.

**IMPORTANCE:**

Green seaweeds of the genus *Ulva* are considered a model system to study microbial interactions with the algal host. Remarkably little is known, however, about viral communities associated with green seaweeds, especially in relation to the health of the host. In this study, we characterized the viral communities associated with healthy and bleached *Ulva*. Our findings revealed the presence of 20 putative novel viruses associated with *Ulva*, encompassing both DNA and RNA viruses. The majority of these viruses were found to be especially abundant in bleached *Ulva* specimens. This is the first step toward understanding the role of viruses in the ecology and aquaculture of this green seaweed.

## INTRODUCTION

Viruses are widespread and abundantly present in the marine environment (1). In fact, with millions of virus-like particles per milliliter of seawater and an estimated 10^23^ viral infections occurring every second, viruses are the most abundant life-form in the ocean (2). They play a fundamental role in ecological processes as drivers of biogeochemical cycles and microbial community compositions (2, 3). Viral infections kill an estimated 20% of the marine microbial biomass per day and are causative agents of high mortality rates in heterotrophic and autotrophic plankton blooms (4, 5). For example, viral lysis can quickly stop bloom formations of the cosmopolitan coccolithophore *Emiliania huxleyi* (Prymnesiophyceae), whose calcite shells constitute around 1/3 of the total marine CaCO_3_ production (6). Viral infections also potentially introduce new genetic information into the infected organism, and viruses, as a whole, comprise an untapped reservoir of genetic diversity (2). Although our knowledge of the impact of viruses in marine environments is increasing, remarkably little is known about viral diversity associated with specific groups of eukaryotes, such as marine macroalgae.

**Fig 1:**
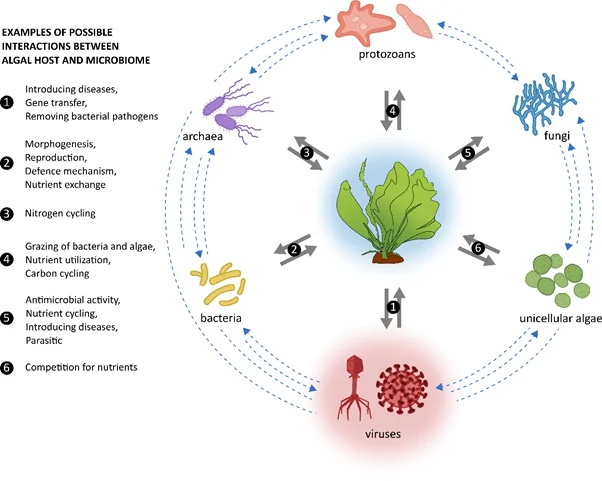
Possible roles and relationships between the algal host, viruses, and the other components of the microbiome. Image concept based on Peixoto et al. (14).

Marine macroalgae (seaweeds) occur worldwide from tropical to arctic coastal ecosystems. As ecosystem engineers and foundation species, they provide food, shelter, and habitat for a wide variety of marine life and are important contributors to total primary productivity (7). In addition, approximately 30% of the global aquaculture production is derived from seaweeds (8). While it becomes increasingly clear that microbes significantly impact their seaweed host, we are largely ignorant about viruses in macroalgae (Fig. 1). Likely the most studied seaweeds with regard to viral infections are the brown, filamentous species belonging to the Ectocarpales. Viral-like particles were first observed in *Ectocarpus siliculosus* laboratory cultures that showed a defect in gametangium (reproductive structures) formation (9). These hexagonal particles were released in the culture medium after the host cells burst and were able to infect healthy cultures. Similar viral particles, identified as phycodnaviruses, were later observed in *Kuckuckia, Hincksia*, and *Feldmannia* (10). More recently, double-stranded DNA (dsDNA) and single-stranded DNA (ssDNA) viruses were found to be associated with several brown macroalgal kelp species (11, 12), and the first RNA virome characterization was completed by Lachnit et al. (13) on a red alga, *Delisea pulchra*. To the best of our knowledge, viral communities of green macroalgae have not been characterized before.

Although prior studies showed that macroalgae harbor a wide diversity of viruses, the biology and ecology of seaweed viruses are poorly understood. Especially the role of viruses in the health and diseases of seaweeds remains a black box (15). The *E. siliculosus* virus 1 (EsV-1 virus) is the best-studied organism, as the virus has been isolated and its genome has been sequenced. The virus is considered pathogenic, as infected *Ectocarpus* thalli become partially or fully sterile (16). In *Feldmannia*, photosynthetic performance is significantly reduced in infected individuals (17). In red seaweeds, only a single disease has been causatively linked to a virus—green spot disease in *Pyropia*—but the viral genome has not been sequenced (18). In brown seaweeds, bleached kelp has been associated with elevated levels of Circular Rep-Encoding Single-Stranded (CRESS) DNA viruses (12), and similarly, bleaching in the green seaweed *Ulva* was also hypothesized to result from virus-like particles (19).

Green seaweeds of the genus *Ulva*—commonly known as Sea Lettuce—are ecologically and economically important seaweed species. Their biomass can be used as a sustainable feedstock due to their high protein content or in the context of bioremediation (i.e., removing excess nutrients) and integrated multi-trophic aquaculture systems (20). However, *Ulva* species are notorious for developing extensive blooms known as green tides. These mass accumulation events have been increasingly observed worldwide and profoundly affect the environment due to the resulting anoxic conditions and the release of gaseous sulfur compounds (21, 22). In addition, *Ulva* species are often used to study algal-bacterial interactions and morphogenesis (23, 24). *Ulva* species depend on appropriate bacterial communities for morphological development and in the absence of specific bacterial strains merely grow as a loose callus-like aggregate of cells (25). In addition to morphogenesis, bacteria play an important role in seaweed growth (26), biochemical composition (27), and the settlement of gametes and spores (28, 29). While the *Ulva* holobiont is considered a model system to study microbial interactions with the algal host, nothing is known about associated viral communities.

Characterizing viral communities associated with *Ulva* is the first step toward understanding the role of viruses in the ecology (e.g., the occurrence of green tides) and aquaculture of this green seaweed. In this study, we characterized *Ulva*-associated DNA and RNA viruses from cultivated and natural populations, as well as bleached and healthy thalli, using metagenomic analyses.

## RESULTS

### The *Ulva* virome composition of healthy and bleached specimens

**Fig 2:**
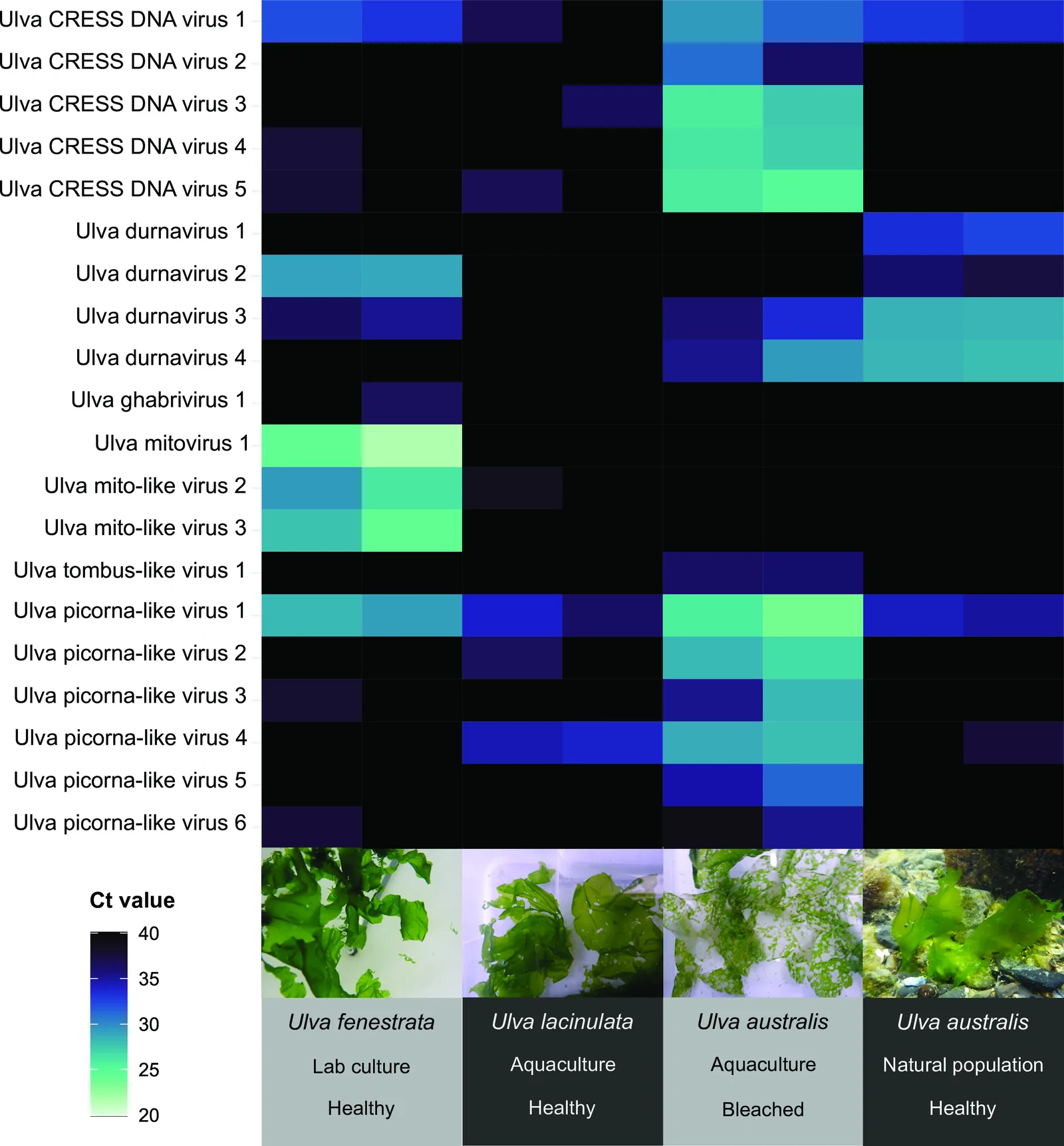
Heatmap showing mean Ct (cycle threshold) qPCR values for the 20 putative new viruses associated with the green seaweed *Ulva*. Low Ct values correspond to high viral load. From each culture or site, 1–2 cm^2^ tissue from two different individuals was sampled.

**Fig 3:**
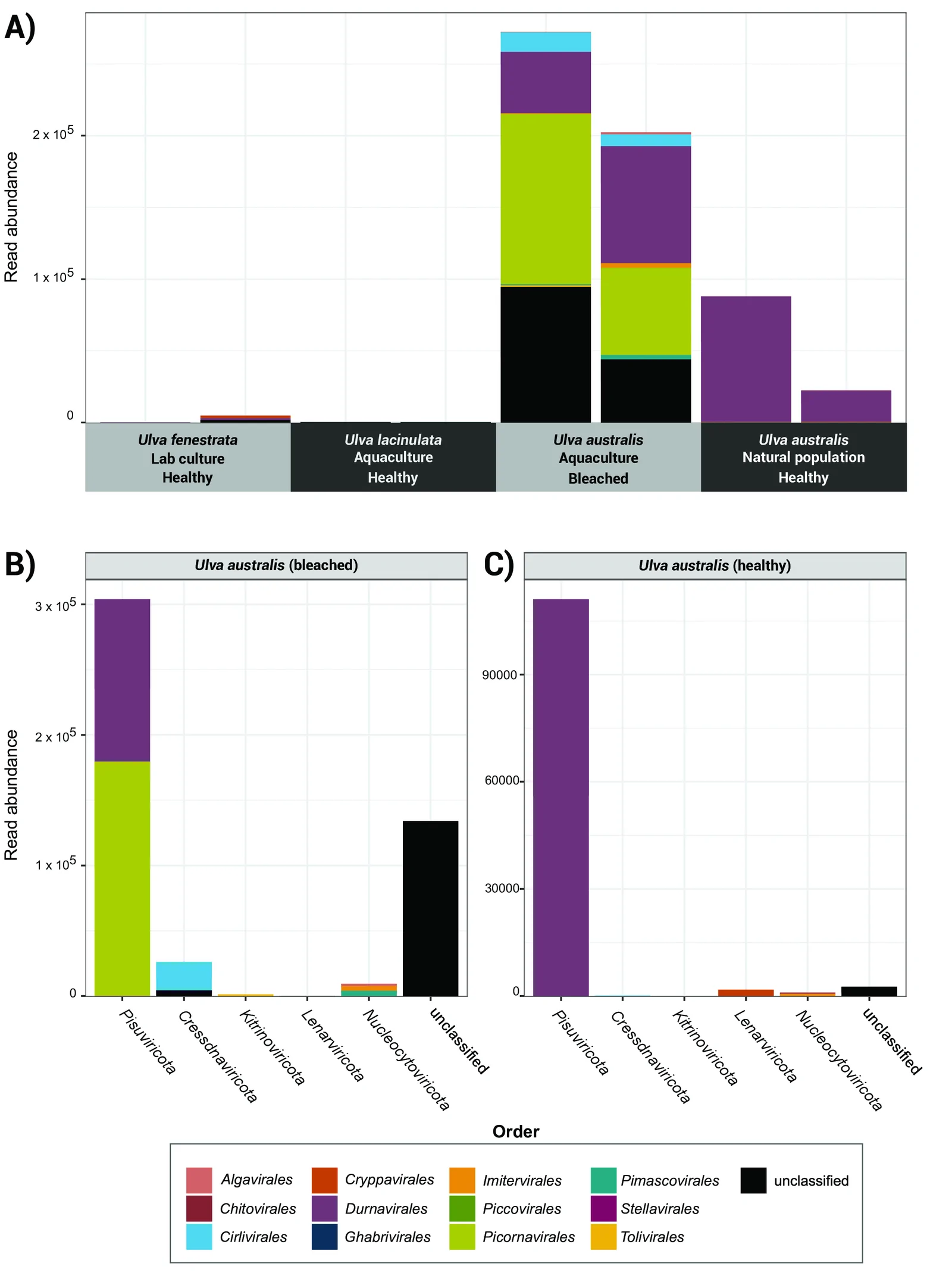
Eukaryotic viral read numbers. (**A**) Total eukaryotic viral reads per sample (*n* = 8; two replicates per sample type). (**B**) Total eukaryotic viral reads from bleached *Ulva australis* viromes that were assigned to the phyla *Pisuviricota*, *Cressdnaviricota*, *Kitrinoviricota*, *Lenarviricota*, *Nucleocytoviricota*, and unclassified viruses (sum of two replicates, total eukaryotic viral reads = 474,578). (**C**) Total eukaryotic viral reads from healthy *Ulva australis* samples from natural populations (sum of two replicates, total eukaryotic viral reads = 110,460). Identifications are based on BLAST with the NCBI database. Colors represent different orders.

A total of 60,952,693 high-quality reads were obtained across all eight samples (Fig. 2), varying between 1,274,891 and 12,926,447 reads per sample. The majority of the reads were assigned to bacteria, the *Ulva* host, and prokaryotic viruses (i.e., phages that infect prokaryotes). A total of 591,075 reads—1% of the total read number—across all eight samples were assigned to eukaryotic viruses (i.e., viruses that infect eukaryotes). The bleached *Ulva australis* samples had the highest number of eukaryotic viral reads (replicate 1 = 272,229 reads; replicate 2 = 202,349 reads), followed by the natural and presumably healthy *U. australis* populations (replicate 1 = 88,101 reads; replicate 2 = 22,359 reads; Fig. 3A). The healthy aquaculture samples (*Ulva fenestrata* and *Ulva lacinulata*) contained the least reads assigned to eukaryotic viruses (varying between 216 and 4,857 viral reads; Fig. 3A).

Across all samples, 144 unique eukaryotic virus contigs were identified. Most reads were assigned to viral contigs classified as belonging to the phyla *Pisuviricota* (70% of the eukaryotic viral reads) and *Cressdnaviricota* (4.4% of the eukaryotic viral reads). However, a large proportion of the viral contigs could not be classified at phylum level (i.e., contigs that matched uncultured marine viruses in the NCBI nr database), accounting for 23% of the eukaryotic viral reads. Within the *Pisuviricota*, healthy and bleached samples contained high read numbers of *Durnavirales* (109,420 reads in healthy samples vs 124,339 reads in the bleached samples; Fig. 3B and C). The bleached samples also contained many reads mapping to contigs belonging to the *Picornavirales* (179,476 reads; Fig. 3B). These results are based on the closest match in the NCBI database. However, the similarity (percentage identity) to the closest match is, in many cases, very low, and the identifications should therefore be treated with caution (Table 1). For example, based on the initial BLAST results, 3.7% of the eukaryotic viral reads were assigned to the order *Cirlivirales* (*Cressdnaviricota*). Our phylogenetic analyses (see section Phylogenetic analyses of putative new viruses), however, indicate that *Ulva*-associated CRESS DNA viruses likely belong to undescribed orders and families within the *Cressdnaviricota*.

**TABLE 1:** BLASTx results of the 20 putative new virus-like sequences against the NCBI nr database

Putative new virus	GenBank accession number	Contig nucleotide length (bp)	BLASTx hit GenBank accession	%ID with BLAST hit	e-Value	BLASTx hit organism	BLASTx hit taxonomy	BLASTx hit host
Ulva CRESS DNA virus 1	OP924572	3,208	AXH74985.1	47.83	3.00E-84	CRESS virus sp.	*Cressdnaviricota*	Minnow (freshwater fish)
Ulva CRESS DNA virus 2	OP924573	2,160	YP009163922.1	55.62	7.00E-68	Gammarus sp. amphipod associated circular virus	*Circoviridae*	*Gammarus* sp. (amphipod)
Ulva CRESS DNA virus 3	OP924574	2,668	YP009551434.1	44.19	1.00E-24	Mosquito VEM virus SDRBAJ	Unclassified DNA virus	Mosquito
Ulva CRESS DNA virus 4	OP924575	2,402	YP009126884.1	46.44	2.00E-78	Avon-Heathcote Estuary associated circular virus 5	*Circoviridae*	*Austrovenus stutchburyi* (marine bivalve)
Ulva CRESS DNA virus 5	OP924576	3,939	YP009553245	29.8	2.00E-05	Lolium perenne-associated virus	Unclassified DNA virus	*Lolium perenne* (plant)
Ulva durnavirus 1	OP924591	2,971	QOW97231.1	44.49	4.00E-60	Amalga-like boulavirus	*Partitiviridae*	*Kraftionema allantoideum* (microalga)
Ulva durnavirus 2	OP924592	2,730	QOW97231.1	41.78	3.00E-50	Amalga-like boulavirus	*Partitiviridae*	*Kraftionema allantoideum* (microalga)
Ulva durnavirus 3	OP924593	3,198	QOW97231.1	40.09	1.00E-39	Amalga-like boulavirus	*Partitiviridae*	*Kraftionema allantoideum* (microalga)
Ulva durnavirus 4	OP924594	1,909	QOW97235.1	25.63	6.00E-20	Partiti-like lacotivirus	*Partitiviridae*	*Ostreobium* sp. (microalgae)
Ulva ghabrivirus 1	OP924595	2,825	BBZ90078.1	30.81	2.00E-76	Diatom RNA virus 1	Unclassified *Riboviria*	*Melosira* sp. (diatom)
Ulva mitovirus 1	OP924596	2,200	AZG04294.1	35.6	1.00E-59	Hymenoscyphus fraxineus mitovirus 1	*Mitoviridae*	*Hymenoscyphus albidus* (fungus)
Ulva mito-like virus 2	OP924597	2,513	APG77166.1	43.61	6.00E-50	Shahe narna-like virus 6	Unclassified *Riboviria*	Freshwater isoptera
Ulva mito-like virus 3	OP924598	2,539	QOW97242.1	28.6	2.00E-45	Mito-like babylonusvirus	Unclassified *Riboviria*	*Ostreobium* sp. (microalgae)
Ulva tombus-like virus 1	OP924599	3,717	YP009337737.1	42.79	3.00E-103	Hubei tombus-like virus 6	Unclassified *Riboviria*	Myriapoda
Ulva picorna-like virus 1	OP924600	8,432	AVP71827.1	27.01	8.00E-56	Macrobrachium rosenbergii dicistrovirus 2	*Dicistroviridae*	*Macrobrachium rosenbergii* (shrimp)
Ulva picorna-like virus 2	OP924601	7,847	QKK13171.1	41	2.00E-79	Kummerowia striata dicistrovirus	*Dicistroviridae*	*Kummerowia striata* (plant)
Ulva picorna-like virus 3	OP924602	7,479	QKK82970.1	36.23	0	Trichosanthes kirilowii picorna-like virus	*Picornavirales*	*Trichosanthes kirilowii* (plant)
Ulva picorna-like virus 4	OP924603	8,334	QKK82968.1	31.14	1.00E-53	Trichosanthes kirilowii picorna-like virus	*Picornavirales*	*Trichosanthes kirilowii* (plant)
Ulva picorna-like virus 5	OP924604	10,369	YP009330008.1	28.68	2.00E-41	Changjiang crawfish virus 4	Unclassified *Riboviria*	Crayfish
Ulva picorna-like virus 6	OP924605	7,970	YP009336663.1	28.98	3.00E-75	Wenling picorna-like virus 6	Unclassified *Riboviria*	Crustacean

### Phylogenetic analyses of putative new viruses

In total, we identified 20 putative new (near complete) viral sequences using a standard sequence similarity search against the NCBI nr reference database (Table 1). The presence or absence of the 20 putative new viruses was verified by qRT-PCR (quantitative real-time PCR) in each of the samples. The putative new viruses exhibited low sequence similarity to existing replicase amino acid sequences (Rep in the case of DNA viruses and RdRp in the case of RNA viruses), with amino acid percentage similarities to the closest matching sequence ranging from 25.6% to 55.6%. They primarily belonged to ssDNA viruses, dsRNA viruses, or single-stranded positive-sense RNA viruses [ssRNA(+)]. Together they represented 82% of the eukaryotic viral reads. The qPCR assay showed that Ulva CRESS DNA viruses and Ulva picorna-like viruses were especially abundant in bleached *Ulva* specimens, while being absent or present in very low numbers (high cycle thresholds) in the healthy samples (Fig. 2; Fig. S1). Mitoviruses and mito-like viruses were especially abundant in the *U. fenestrata* lab culture, and Ulva durnaviruses were mainly present in both healthy and bleached *U. australis* (Fig. 2; Fig. S1).

The putative new viruses are most likely exogenous, as the same viral contigs were found in each of the duplicated samples, and the viral contigs assembled without attachment of *Ulva* genome fragments on either side. In addition, many of the putative new viruses were absent in the healthy *Ulva* samples, which would be unexpected if the contigs belonged to endogenous viral elements. Finally, we extracted total DNA and RNA following the NetoVir protocol, which is optimized for virus-like particles (endogenous viral elements are not protected by a capsid and are therefore not extracted using NetoVir) (30).

### CRESS DNA viruses

**Fig 4:**
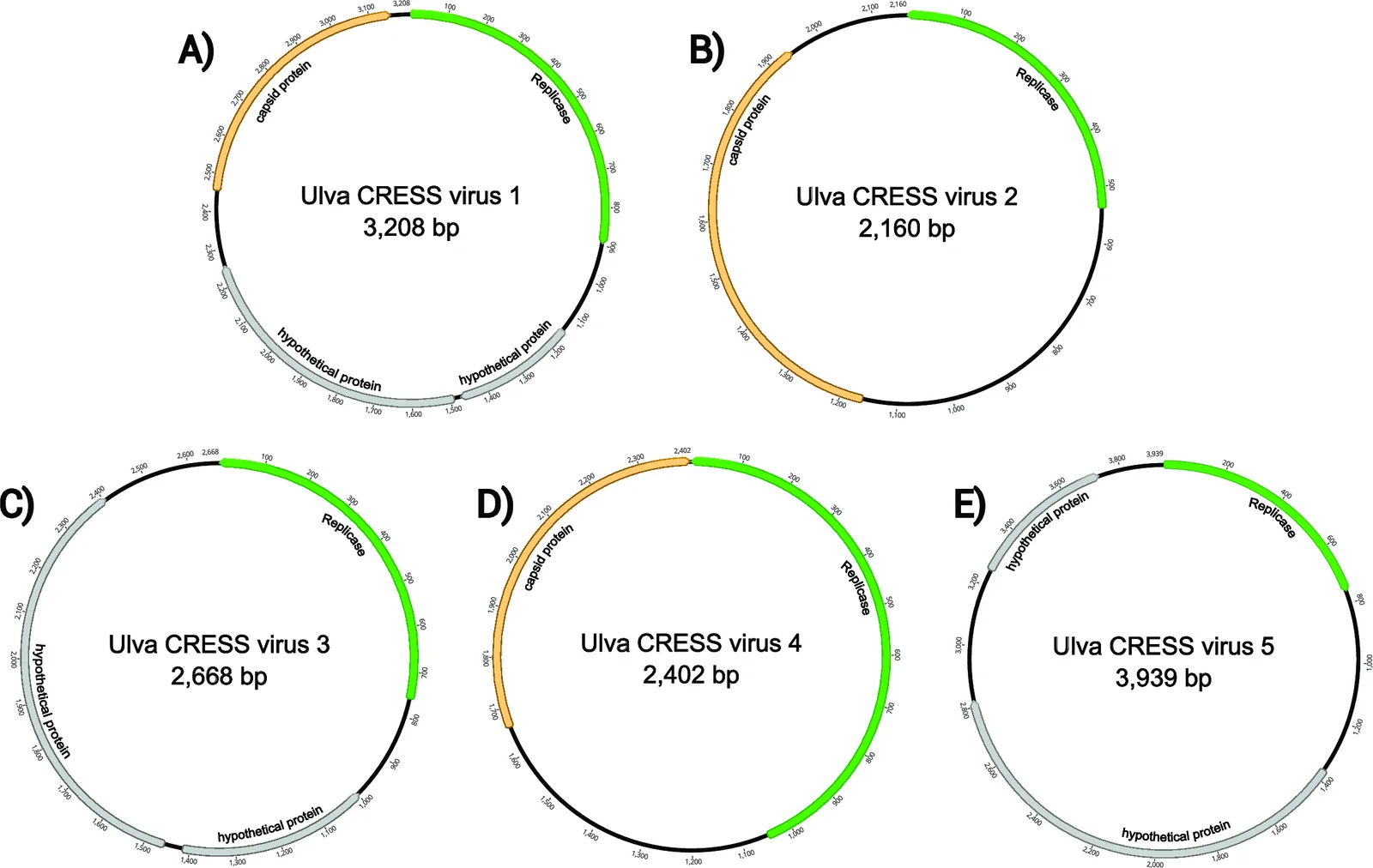
The genome organization of the five Ulva CRESS viruses, including replicase and capsid protein genes. Open reading frames (ORFs) were predicted using ORFfinder (https://www.ncbi.nlm.nih.gov/orffinder/).

Five viral assembled contigs associated with *Ulva* exhibited similarity to CRESS DNA viruses (*Cressdnaviricota*) belonging to the classes *Arfiviricetes* and *Repensiviricetes* (Fig. 4). CRESS DNA viruses have small, circular genomes, with a genome size often ranging from 1.0 to 6.5 kb (31). Many of these genomes contain only two major Open Reading Frames (ORFs) that encode the replication initiator protein (Rep) and the capsid protein (Cap). CRESS DNA viruses are believed to infect a wide variety of hosts, including mammals, birds, insects, fungi, diatoms, and plants (32).

**Fig 5:**
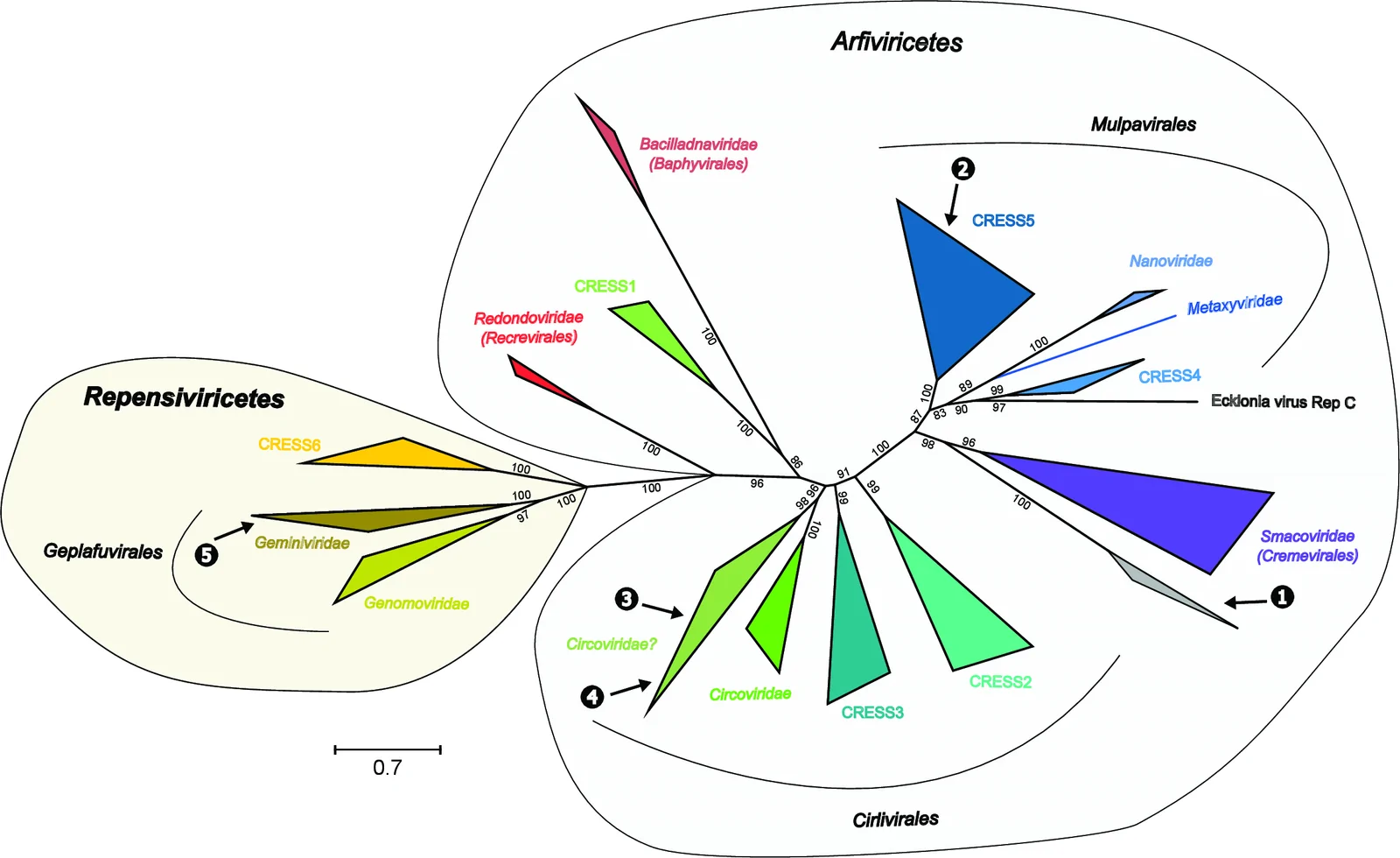
Unrooted maximum likelihood phylogenetic tree of Rep proteins from CRESS DNA viruses estimated using IQ-Tree with ultrafast bootstrap replicates (values below 70 are not displayed). Closely related sequence groups are collapsed into triangles, whose side lengths are proportional to the distances between the closest and farthest leaf nodes. The locations of the *Ulva*-associated CRESS DNA viruses are marked with numbered arrows. The numbers correspond to Ulva CRESS DNA virus 1–5. Branch lengths are scaled according to the number of amino acid substitutions per site.

**Fig 6:**
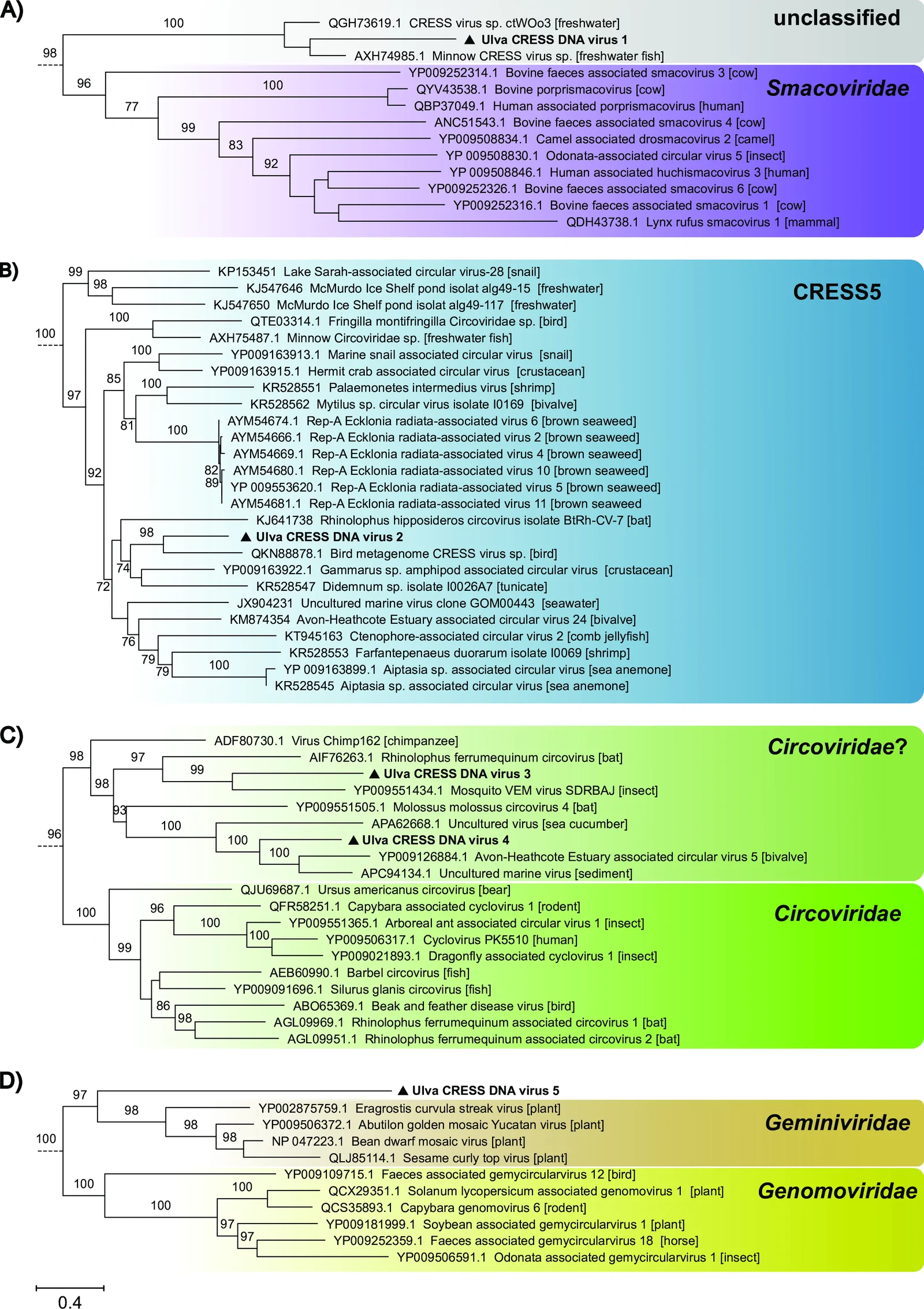
Details of the phylogeny of CRESS DNA viruses based on the Rep protein. (**A**) The *Smacoviridae* and unclassified clade, (**B**) CRESS5 group, (**C**) *Circoviridae*, and (**D**) *Repensiviricetes* (*Genomoviridae* and *Geminiviridae*). Newly discovered viruses from *Ulva* spp. are highlighted in bold. Each viral sequence’s putative eukaryotic host (or sample habitat) is displayed in brackets. Maximum likelihood tree estimated using IQ-Tree with ultrafast bootstrap replicates (values below 70 are not displayed). The tree is mid-point rooted. Branch lengths are scaled according to the number of amino acid substitutions per site.

The five *Ulva*-associated CRESS DNA viruses grouped with very diverse orders and families (Fig. 5). Four contigs belonged to the *Arfiviricetes*, and one was positioned within the *Repensiviricetes* clade. Ulva CRESS DNA virus 1 was placed in an unidentified clade most closely related to the family *Smacoviridae* in the order *Cremevirales* (Fig. 6A). Ulva CRESS DNA virus 2 was located within the CRESS5 clade (33). This group also contained Rep-A viruses associated with *Ecklonia radiata* (a brown algal kelp species), as well as many other marine viruses associated with, amongst others, tunicates, amphipods, ctenophores, and marine snails (Fig. 6B). Ulva CRESS DNA viruses 3 and 4 were positioned in a clade most closely related to the family *Circoviridae* and most likely belong to the order *Cirlivirales*. This clade also contained several viruses isolated from marine organisms and environments (Fig. 6C). Ulva CRESS DNA virus 5 grouped between the families *Geminiviridae* and *Genomoviridae* (*Geplafuvirales* and *Repensiviricetes*; Fig. 6D). *Geminiviridae* and *Genomoviridae* viruses are mostly known to infect plants and fungi, respectively.

### Durna-like dsRNA viruses

Four putative new viruses were found to belong to the *Durnavirales*. This order currently includes six families (*Partitiviridae*, *Amalgaviridae*, *Picobirnaviridae*, *Hypoviridae*, *Fusariviridae*, and *Curvulaviridae*). Viruses belonging to the *Durnavirales* are mostly known to infect plants, fungi, and protists (34). The RdRp amino acid pairwise identity between the four *Ulva*-associated durnaviruses was relatively low but variable (14.7%–61.8%). The ICTV (International Committee on Taxonomy of Viruses) currently uses 85% RdRp amino acid sequence identity as a demarcation criterion for the genus *Amalgavirus* (35). We therefore assume that the four *Ulva*-associated sequences each represent a new virus genus or higher taxonomical rank.

**Fig 7:**
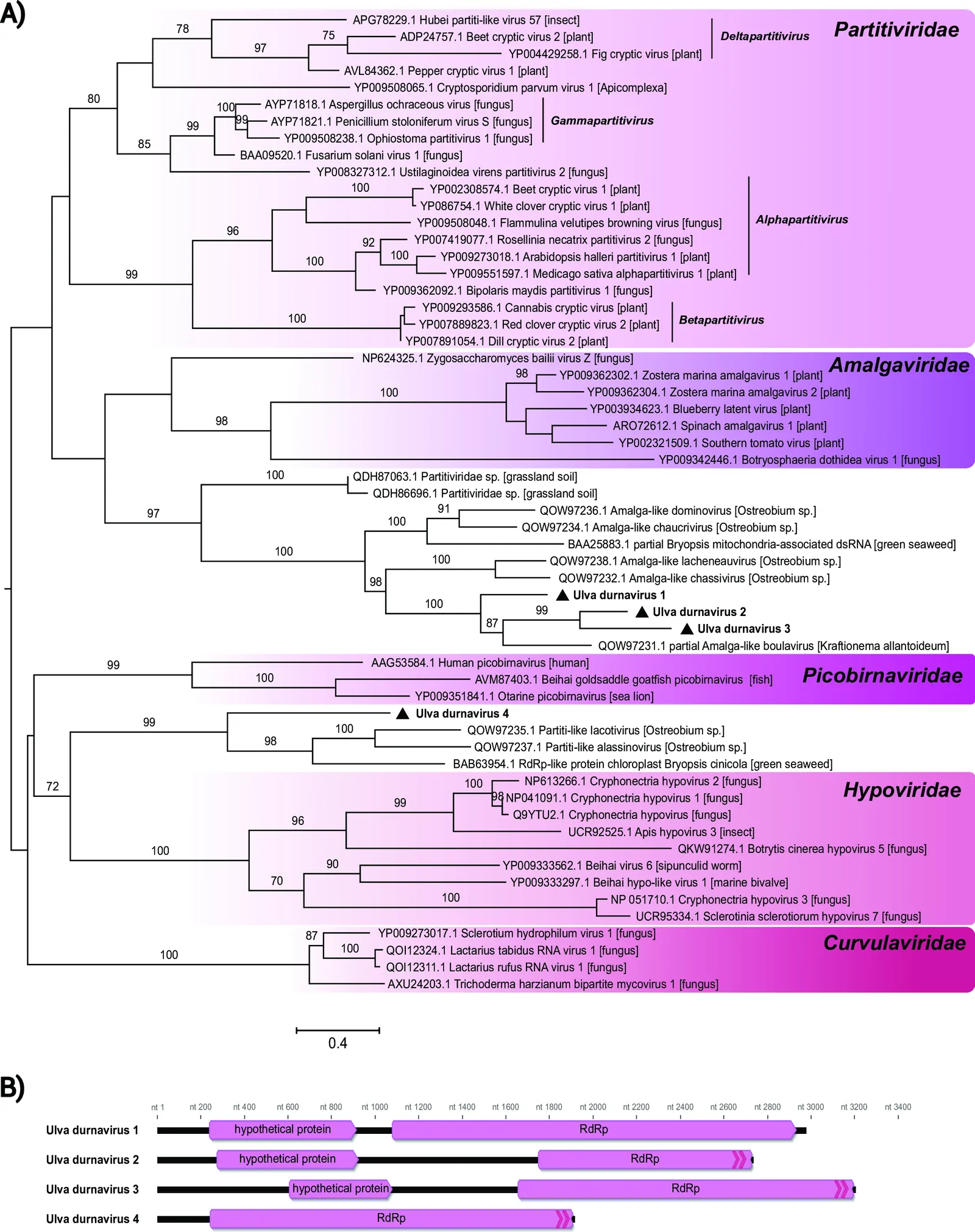
Phylogeny and genome organization of the Ulva durnaviruses. (**A**) Phylogentic tree of the order *Durnavirales* based on the RdRp protein. Newly discovered viruses from *Ulva* spp. are highlighted in bold and indicated with a triangle. Each viral sequence’s putative eukaryotic host (or sample habitat) is displayed in brackets. Maximum likelihood tree estimated using IQ-Tree with ultrafast bootstrap replicates (values below 70 are not displayed). The tree is mid-point rooted. Branch lengths are scaled according to the number of amino acid substitutions per site. (**B**) Genome organization, including RNA-dependent RNA polymerase (RdRp) genes. Open Reading Frames (ORFs) were predicted using ORFfinder (https://www.ncbi.nlm.nih.gov/orffinder/).

Three of the newly described viral sequences exhibited RdRp amino acid sequence similarity to Amalga-like sequences (relatives of the *Amalgaviridae*; Fig. 7A). *Amalgaviridae* have linear dsRNA genomes of ~3.5 kb containing two overlapping ORFs (34). The contig lengths of the putative new viruses varied from 2,730 to 3,198 nt, and they likely represent (near) complete genomes (Fig. 7B). Each genome contained two ORFs, of which the largest one coded for the RdRp and the smaller one did not exhibit similarity to known proteins (Fig. 7B). Ulva durnaviruses 1, 2, and 3 were most closely related to Amalga-like boulavirus isolated from *Kraftionema allantoideum* (a microalga belonging to the Ulvophyceae; Fig. 7A). The other viruses in this clade were related to the *Ostreobium* sp. and *Bryopsis* mitochondria-associated dsRNA viruses (both hosts belong to the Ulvophyceae). The latter was initially described as dsRNA associated with mitochondria in the green macroalga *Bryopsis cinicola* (36) but likely also represents a virus (37). Charon et al. (38) hypothesized that these viruses formed a Ulvophyceae-infecting viral clade.

Ulva durnavirus 4 was positioned within a clade most closely related to the *Hypoviridae*, together with Partiti-like viruses associated with *Ostreobium* sp. (Ulvophyceae; Fig. 7A). The contig of Ulva durnavirus 4 only contained one ORF (coding for RdRp; Fig. 7B). As this was a relatively small contig (1,909 nt), it is likely that the genome of this virus is segmented (or incomplete), similar to viruses belonging to the *Partitiviridae* and *Picobirnaviridae* (39). Linking additional genomic segments for this virus, however, is not possible based solely on metagenomics analysis.

### *Ghabrivirales* (linear dsRNA viruses)

**Fig 8:**
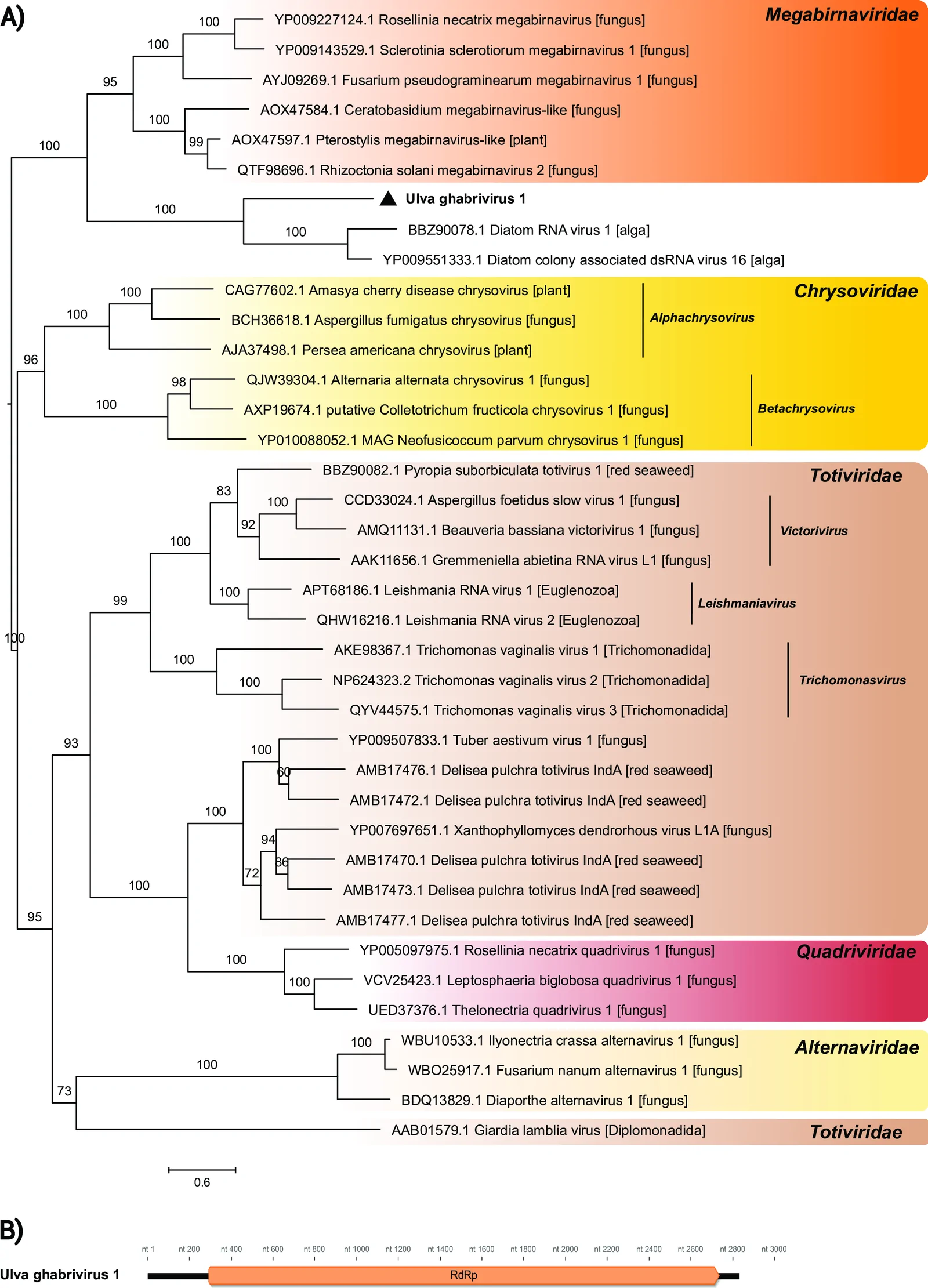
Phylogeny and genome organization of Ulva ghabrivirus 1. (**A**) Phylogenetic tree of the *Ghabrivirales* based on the RdRp protein. The newly discovered Ulva ghabrivirus is highlighted in bold and indicated with a triangle. Each viral sequence’s putative eukaryotic host (or sample habitat) is displayed in brackets. Maximum likelihood tree estimated using IQ-Tree with ultrafast bootstrap replicates (values below 70 are not displayed). The tree is mid-point rooted. Branch lengths are scaled according to the number of amino acid substitutions per site. (**B**) Genome organization, including the RNA-dependent RNA polymerase (RdRp) gene. The genomes of the *Ghabrivirales* are segmented, and it is therefore likely that the contig of Ulva ghabrivirus 1 only represents a partial genome. Open Reading Frames (ORFs) were predicted using ORFfinder (https://www.ncbi.nlm.nih.gov/orffinder/).

One *Ulva*-associated contig displayed RdRp amino acid sequence similarity to two diatom-associated viruses (31%–32%). These viruses form a clade closely related to the *Megabirnaviridae*, a predominantly fungi-infecting family within the order *Ghabrivirales* (Fig. 8A) (40). Other families within the *Ghabrivirales* include the *Chrysoviridae* (infecting plants and fungi), the *Quadriviridae* (infecting fungi), and the *Totiviridae* (infecting fungi and red macroalgae) (13, 41). It is unclear whether the *Ulva*- and diatom-associated viruses form a new family within the *Ghabrivirales* or represent members of the *Megabirnaviridae*.

The genomes of the *Megabirnaviridae*, *Chrysoviridae*, and *Quadriviridae* are segmented, containing two to seven segments ranging from 2.7 to 9 kb in size (41 – 43). It is therefore likely that the contig of Ulva ghabrivirus 1 (2,825 nt, containing 1 ORF coding for RdRp) only represents a partial genome (Fig. 8B).

### Mito-like ssRNA(+) viruses

**Fig 9:**
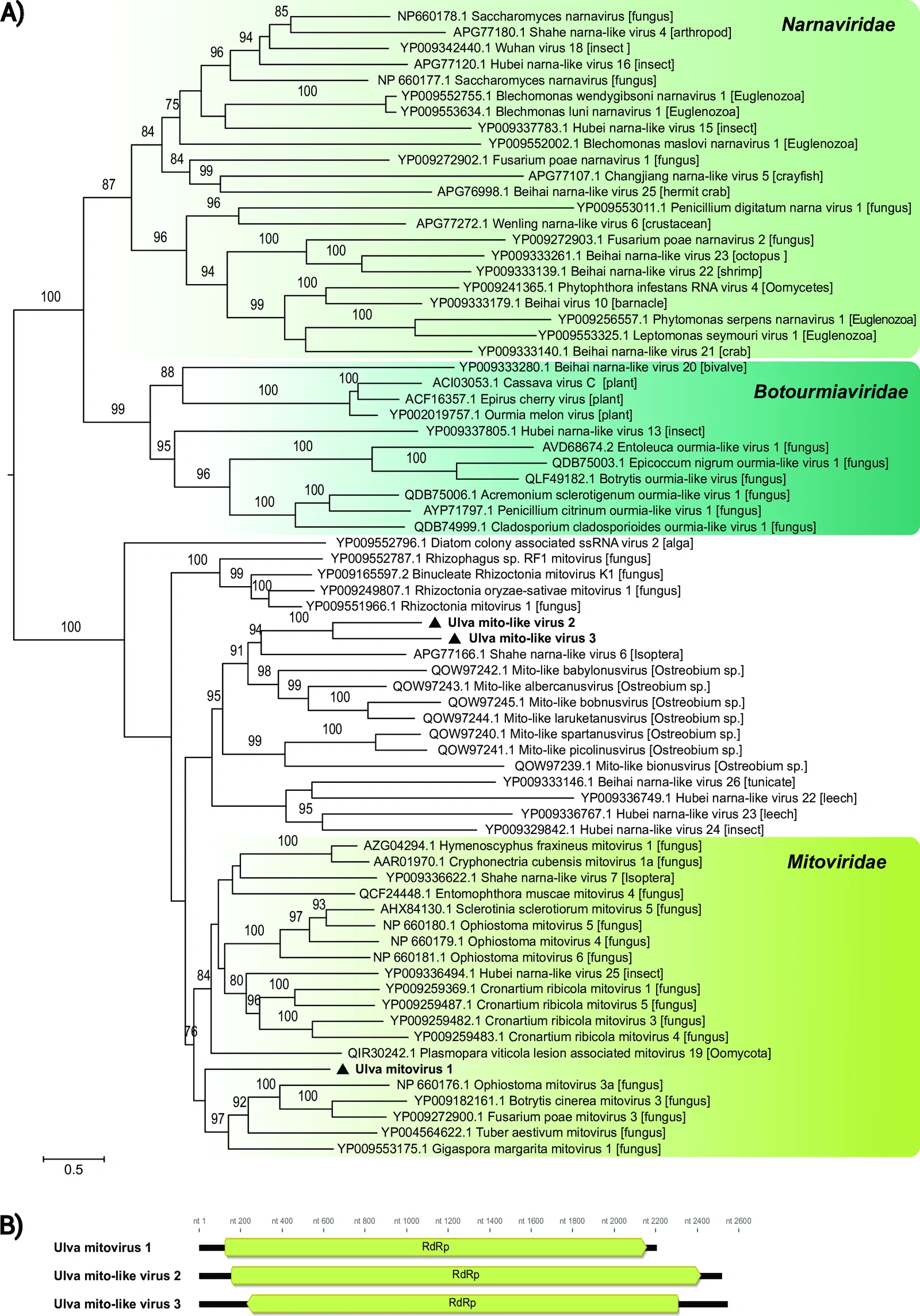
Phylogeny and genome organization of Ulva mitoviruses and mito-like viruses. (**A**) Phylogenetic tree of the *Narnaviridae*, *Botourmiaviridae*, and *Mitoviridae* based on the RdRp protein. Newly discovered viruses from *Ulva* spp. are highlighted in bold and indicated with a triangle. Each viral sequence’s putative eukaryotic host (or sample habitat) is displayed in brackets. Maximum likelihood tree estimated using IQ-Tree with ultrafast bootstrap replicates (values <70 are not displayed). The tree is mid-point rooted. Branch lengths are scaled according to the number of amino acid substitutions per site. (**B**) Genome organization, including RNA-dependent RNA polymerase (RdRp) genes. Open Reading Frames (ORFs) were predicted using ORFfinder (https://www.ncbi.nlm.nih.gov/orffinder/).

Three viral contigs associated with *Ulva* were related to the *Mitoviridae* (Fig. 9A). *Mitoviridae* is currently the only family recognized within the order *Cryppavirales*. Viruses of this family have small genomes of up to 3 kb containing a single ORF encoding an RdRp gene (44). *Mitoviridae* have mostly been reported from fungi but, to a lesser degree, have been found associated with insects (45, 46), algae (38), and plants (47). The contig size of the viruses sequenced in this study varied between 2,200 and 2,539 bp, which is within the standard size ranges of viruses belonging to the family *Mitoviridae*. All three viral sequences contained a single ORF encoding for the RdRp domain (pfam05919; Fig. 9B).

Ulva mitovirus 1 was positioned within a clade formed by the family *Mitoviridae*. This clade is dominated by viruses infecting fungi. Ulva mito-like virus 2 and 3 were closely related to each other (42% RdRp similarity), and seven mito-like viruses are associated with the green microalga *Ostreobium* sp. (Ulvophyceae; ranging from 25% to 45% similarity). This clade possibly represents a new genus within the family *Mitoviridae* or a new family within the order *Cryppavirales*.

### Tombus-like ss(+)RNA viruses

**Fig 10:**
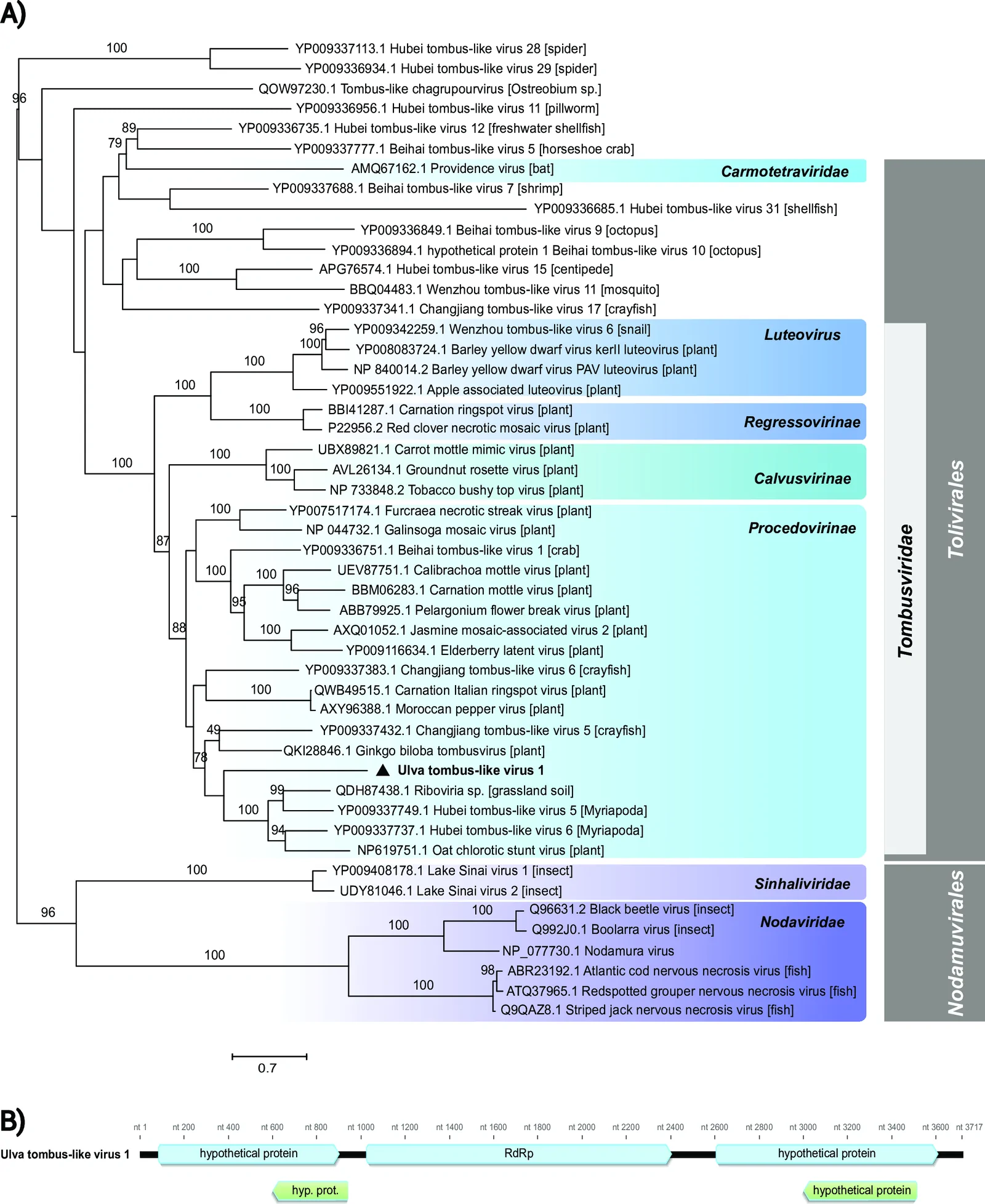
Phylogeny and genome organization of Ulva tombus-like virus 1. (**A**) Phylogenetic tree of the *Tolivirales* (*Tombusviridae* and *Carmotetraviridae*) and the *Nodamuvirales* based on the RdRp protein. The newly discovered Ulva tombus-like virus is highlighted in bold and indicated with a triangle. Each viral sequence’s putative eukaryotic host (or sample habitat) is displayed in brackets. Maximum likelihood tree estimated using IQ-Tree with ultrafast bootstrap replicates (values <70 are not displayed). The tree is mid-point rooted. Branch lengths are scaled according to the number of amino acid substitutions per site. (**B**) Genome organization, including RNA-dependent RNA polymerase (RdRp) genes. Open Reading Frames (ORFs) were predicted using ORFfinder (https://www.ncbi.nlm.nih.gov/orffinder/).

One contig, Ulva tombus-like virus 1, exhibited similarity to members of the *Tombusviridae* [a plant-infecting family of ssRNA(+) viruses] and clustered within the subfamily *Procedovirinae* (Fig. 10A). Viral genomes of members of the *Tombusviridae* range in size from 3.7 to 4.8 kb and contain 4–6 ORFs (48). These ORFs encode replication-associated proteins, movement proteins, and capsid proteins. Ulva tombus-like virus 1 was represented by a 3.7 kb contig that included five ORFs (Fig. 10B). The largest ORF encoded the RdRp gene. The remaining four ORFs did not match with any reference protein sequences.

Ulva tombus-like virus 1 was most closely related to tombus-like viruses associated with grassland soil, Myriapoda, and Oat chlorotic stunt virus (a plant pathogen). RdRP amino acid similarity between Ulva tombus-like virus 1 and the latter three viruses ranged from 22% to 43%, which is well below the species demarcation criteria currently recognized within the different genera of the *Tombusviridae* by the ICTV (57%–93%) (49).

### Picorna-like ss(+)RNA viruses

Six *Ulva*-associated viruses were classified as belonging to the order *Picornavirales*. The *Picornavirales* is a diverse order, currently containing nine families. Members of the *Picornavirales* are known to infect invertebrates (families *Dicistroviridae*, *Iflaviridae*, *Polycipiviridae*, *Noraviridae*, and *Solinviviridae*), vertebrates (family *Picornaviridae* and *Caliciviridae*), plants (family *Secoviridae*), and algae (family *Marnaviridae*; 48; https://ictv.global/taxonomy). They also occur widely and abundant in environments such as oceans (50), rivers (51), and lakes (52). Members of the *Picornavirales* have mono- or bipartite genomes (7,000–12,500 nt) encoding at least one polyprotein. These polyproteins include a replication block with three domains: a superfamily III helicase (Hel), a proteinase (Pro), and a superfamily I RdRp (Pol) (53).

**Fig 11:**
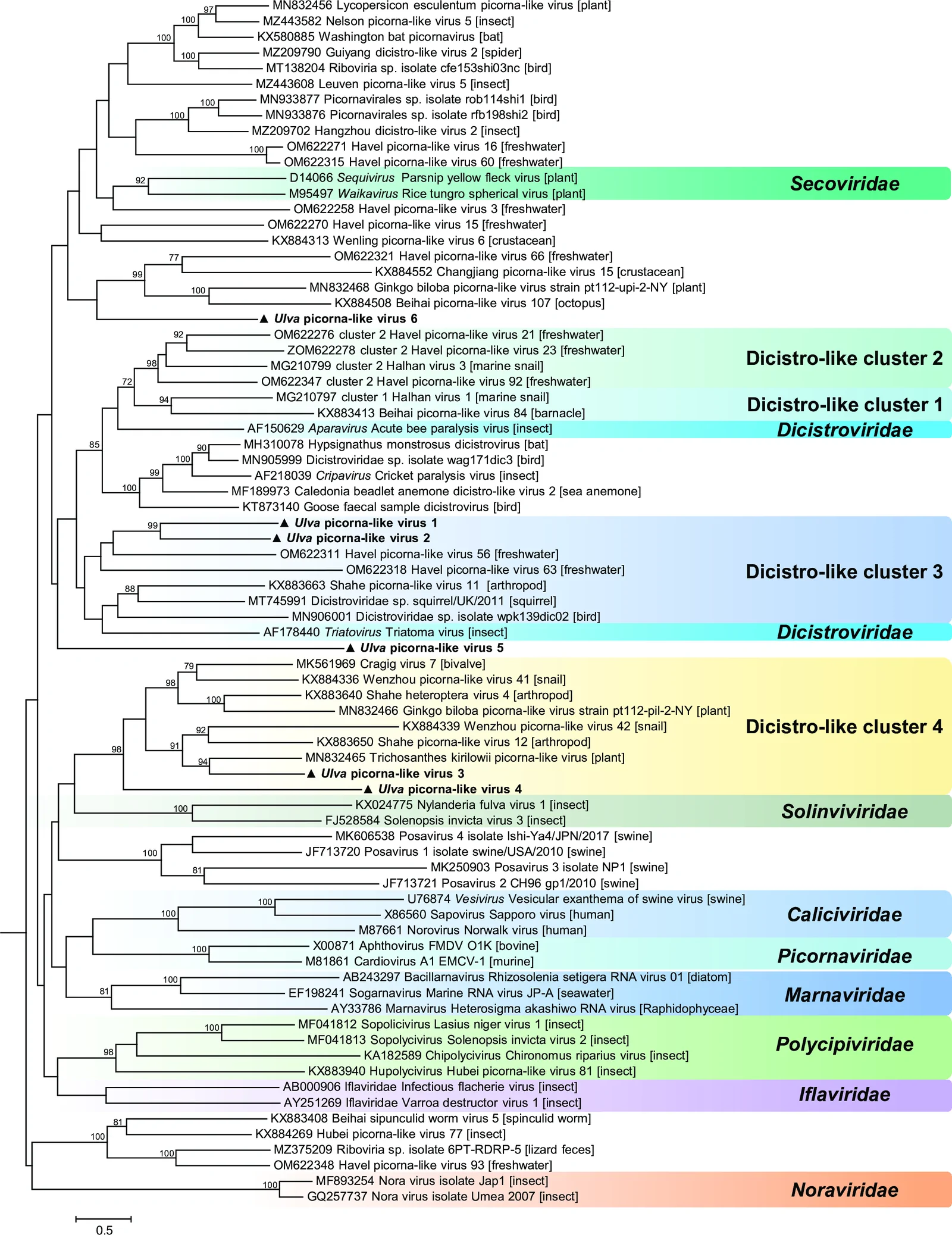
Phylogeny of the *Picornavirales* based on the proteinase/polymerase gene region. Newly discovered viruses from *Ulva* spp. are highlighted in bold. Each viral sequence’s putative eukaryotic host (or sample habitat) is displayed in brackets. Maximum likelihood tree estimated using IQ-Tree with ultrafast bootstrap replicates (values <70 are not displayed). The tree is mid-point rooted. Branch lengths are scaled according to the number of amino acid substitutions per site.

**Fig 12:**
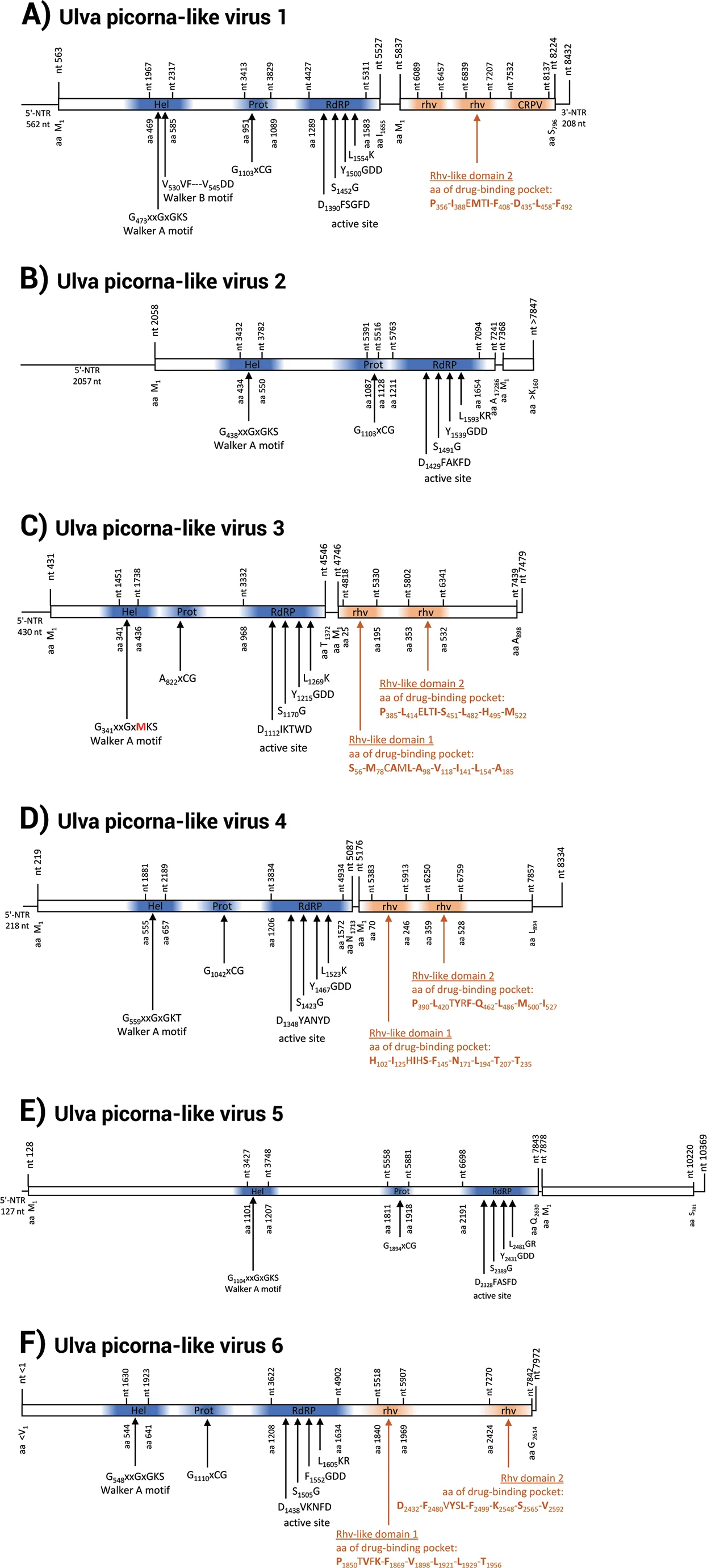
The genome organization and conserved picornaviral motifs of the six Ulva picorna-like viruses. Arrows indicate conserved motifs (Walker A and B motifs, proteinase active site motif, polymerase motifs, and drug-binding pockets of capsid proteins). Rhinovirus-like (rhv-like) domains, including conserved amino acids of the drug-binding pocket, were also identified. Conserved motifs were identified with Pfam and Phyre2.

Ulva picorna-like viruses 1 and 2 were related to each other and members of the *Dicistroviridae* (Fig. 11). The closest relatives were Havel picorna-like virus 56 and Havel picorna-like virus 63 (both isolated from a river), which belong to a Dicistro-like cluster 3 (51). For Ulva picorna-like virus 1, a near complete, dicistronic genome was obtained, and for Ulva picorna-like virus 2 a partial genome, lacking the 3′end (Fig. 12A and B). Ulva picorna-like virus 2 likely has a dicistronic genome as well, containing two polyproteins.

Ulva picorna-like viruses 3 and 4 clustered within Dicistro-like cluster 4 (51), together with *Trichosanthes kirilowii* picorna-like virus (closest relative), and several other unclassified *Picornavirales* members associated with arthropods, snails, plants and bivalves (Fig. 11). The contig of Ulva picorna-like virus 3 represented an almost complete dicistronic genome, with polyprotein 1 encoding the nonstructural protein and a polyprotein 2 encoding the capsid protein (Fig. 12C). Two capsid protein domains with similarity to the cd00205 protein family (rhv-like) including conserved amino acids of the drug-binding pocket were identified by Pfam. This drug-binding pocket is conserved in the three major capsid proteins of all rhinoviruses and enteroviruses, in the capsid proteins of almost all picornaviruses, and in many capsid proteins of picorna-like viruses, making this a good indication of a jellyroll fold of a capsid protein. The putative helicase exhibited a modified Walker A motif (GxxGxMKS). A proteinase domain was not detected using the PFAM (protein family) conserved domain search tool. However, there was a modified active site sequence (AxCG rather than GxCG). Ulva picorna-like virus 4 likewise had a dicistronic genome containing two polyproteins (Fig. 12D). Both ORFs were separated by only 90 nt, suggesting either an unknown type of internal ribosome entry site or another mechanism of translation initiation, e.g., transcription of subgenomic RNA.

Ulva picorna-like virus 5 was highly divergent (Fig. 11). The first ORF in the dicistronic genome encoded an exceptionally long polyprotein 1 (2,630 aa) with picorna-like helicase, proteinase, and polymerase domains (Fig. 12E). The N-terminal (1,000 aa) shared no similarity to any known protein. Polyprotein 2 (781 aa) did not share similarity to known sequences either but is expected to encode capsid proteins.

Ulva picorna-like virus 6 clustered with several picorna-like viruses isolated from the Havel river (51), and with other unclassified picorna-like viruses associated with crustaceans and insects (Fig. 11). Ulva picorna-like virus 6 had a monocistronic genome encoding a single polyprotein (~2,600 aa) with N-terminal nonstructural proteins (Hel-Prot-RdRp) and C-terminal capsid proteins (two rhv domains with drug binding pockets identified by a conserved domain search; Fig. 12F).

## DISCUSSION

It is becoming increasingly clear that viruses are important components of microbial communities. Their role in seaweed health and disease, however, remains poorly studied. We aimed to characterize the DNA and RNA virosphere of the green seaweed *Ulva*. After analyzing eight samples (originating from three different *Ulva* cultures and a natural population), we identified 20 putative new and divergent viruses, of which the majority was especially abundant in bleached *Ulva* specimens. However, it is important to realise that based on metagenomic data alone, it is not possible to establish whether these newly discovered viruses truly infect green algae (i.e., the source of the sequences does not necessarily represent the actual host) (54). The question, therefore, remains whether some of the newly identified viruses may have been associated with other hosts, e.g., fungi or diatoms. While the cultures used in this study were not axenic, the overwhelming majority of eukaryotic reads in our samples were assigned to the genus *Ulva*, making it likely that most of these 20 putative new viruses indeed infect green seaweeds rather than associated eukaryotic microbes.

### Comparing the *Ulva* virome to other seaweeds and microalgae

This study is the first attempt at characterizing the virosphere of a green macroalga. Viromes of green microalgae have received considerably more attention than their larger counterparts, especially in relation to phytoplankton blooms. The impact of viral lysis on phytoplankton mortality can be tremendous (3), and as most algal viruses have a specific host range, they effectively control bloom dynamics (5). Nucleocytoplasmic large DNA viruses, for example, are known to infect the green unicellular prasinophytes *Micromonas pusilla* and *Ostreococcus tauri* (1, 55), with interactions being strongly affected by environmental factors. For instance, continuous light (24:0 light:dark) increased the maximum virus production rate of prasinovirus MpoV-45T, and higher seawater temperature led to earlier algal cell lysis (56). Phycodnaviruses are also found in association with brown seaweeds, including multiple species of kelp (Laminariales) (11, 57) and filamentous species (Ectocarpales) (10, 58, 59). Overall, 40%–100% of *Ectocarpus* individuals and 35% of kelp individuals collected in Europe were infected with phaeoviruses (57), but their biological and ecological relevance is still unknown. Members of the *Phycodnaviridae* were also present in a small proportion of reads in our data set (in the bleached *Ulva* specimens) based on NCBI blast results, but as these viruses contain relatively large genomes, we did not obtain (near) complete genome assemblies of the *Phycodnaviridae* members in this study.

Although research on microalgal virospheres has primarily focused on DNA viruses, recent studies have also demonstrated a large diversity of algae-associated RNA viruses (38, 60, 61). One of these studies included two Ulvophyceaen hosts, *Kraftionema allantoideum* and an *Ostreobium* sp. (38). Similar to the *Ulva* virosphere, the viral communities of *K. allantoideum* and *Ostreobium* sp. were rich in members of the *Durnavirales*. The Ulvophyceaen viruses formed two separate clades most closely related to the *Amalgaviridae* and *Hypoviridae*. The virome of *Ostreobium* sp. also contained multiple mito-like viruses closely related to the mito-like viruses found here in *Ulva. Picornavirales* were not found in *Ostreobium* and *Kraftionema* cultures (38) but have been sequenced from non-green algae such as *D. pulchra* (Rhodophyta) (13), *Vaucheria litorea* (Xanthophyceae), *Symbiodinium* sp. (Dinophyceae), and *Thalassiothrix antarctica* (Bacillariophyta) (60). These picorna-like viruses, however, all belonged to the family of the *Marnaviridae* (data not shown) and are, therefore, not closely related to the *Picornavirales* members found in the current study. Most viral genomes sequenced from algal hosts represent putative new viruses, often belonging to unidentified families or even orders. We have likely only touched the surface with regard to the diversity of algal viruses.

### High viral load in bleached *Ulva*

The high number of viral reads in bleached *Ulva* specimens especially stands out. Only a handful of studies have shown a link between viruses and diseases in seaweeds (62). Green-spot disease, the most common disease in Korean *Pyropia* seaweed farms, is the only known red seaweed disease confirmed to be caused by a virus (18). This chloroplast-infecting virus PyroV1 causes cellular lysis, resulting in green spots on the purple-red seaweed tissue, eventually leading to lysis of the whole blade. Infection experiments showed that PyroV1 could infect at least three *Pyropia* species (*P. dentata*, *P. tenera*, and *P. yezoensis*), but not more distantly related red seaweeds like *Bostrychia tenuissima* and *Dasysiphonia chejuensis*, displaying host-specificity to a certain extent (18). Interestingly, the *Pyropia* species were only susceptible to infection throughout their gametophyte stage (during which they are blade-shaped), not in their conchocelis phase (tetrasporophytic filamentous growths that are often shell-boring) (18). The latent period (i.e., the time required for the disease to become visible in a newly infected host) was 36–54 hours, meaning the disease could spread quickly within aquaculture facilities. Seaweed aquaculture currently represents 51.3% of the total marine and coastal aquaculture production (63) and is still growing worldwide (64). As most crops are cultivated as monocultures, with a higher vulnerability to disseminating diseases, virus detection may become increasingly important.

In brown seaweeds, CRESS DNA viruses were found to be associated in high abundance with bleached phenotypes of the habitat-forming kelp *E. radiata* (12). These ssDNA viruses belong to the CRESS5 clade, together with other marine-habitat viruses, such as crustaceans, sea anemones, and Ulva CRESS DNA virus 2 from this study (Fig. 5). Our bleached *Ulva* specimens were associated with several CRESS DNA viruses and picorna-like viruses that were not found, or only found in low abundance, in healthy specimens. The highly abundant durnaviruses were also found in the natural, healthy population and are therefore not likely to induce bleaching. It is possible that bleaching in *Ulva* is caused by one or a combination of the CRESS DNA viruses or picorna-like viruses, possibly inducing cell lysis similarly to the chloroplast-infecting virus in *Pyropia*. However, as the viruses were not isolated, an unequivocal link between a virus and the bleaching disease could not be verified. Confirming such a link requires additional studies including the isolation of viruses and infection assays. Bleaching of the *Ulva* thalli may also have other causative agents (e.g., bacteria, fungi, and abiotic stress), simply allowing the proliferation of viruses in the already unhealthy seaweeds.

To our knowledge, diseases in green seaweeds—whether caused by bacteria, fungi, or viruses—have not been reported. Bleaching in *Ulva* has however been observed in the bay of Marseille (Mediterranean sea) (19). Contrary to many other coastal areas, the bay of Marseille does not experience green tides (i.e., mass accumulation events of unattached green seaweeds), despite high nutrient availability. When *Ulva* was collected from Brittany (Atlantic coast of France where green tides frequently occur but no bleaching is observed) and cultivated in Marseille seawater, bleaching was rapidly induced, with the thalli sometimes turning white within 1 day. Bleaching coincided with the observation of virus-like particles in seawater, which led Loret et al. (2020) to hypothesize that a marine virus prevents *Ulva* proliferation and green tides in the bay of Marseille. Green tides also happen frequently in the Yellow Sea in China and are becoming a growing concern (22). A recent study that characterized viral communities in seawater surrounding algal blooms in the coastal waters of the Yellow Sea showed that viral richness and community composition changed throughout the different phases of the green tide. For example, *Phycodnaviridae*—known to infect algal hosts—were found to drastically increase during green tides and in the post-bloom phase, as did the proportion of lytic viruses in general (65). In addition, the viral communities were influenced by other environmental parameters, such as total organic carbon, dissolved oxygen, nutrients, and chlorophyll a concentrations. The above examples emphasize how little we know of seaweed viruses, while the effect of these viruses on ecosystems and aquaculture could be tremendous.

## MATERIALS AND METHODS

### Sample collection and algal cultures

Six *Ulva* tissue samples were collected from three different cultures. The first culture, *U. fenestrata*, was originally collected in Sweden and had been maintained for 1 year at Ghent University at the moment of sampling (*n* = 2; Fig. 2). Cultures were maintained in 50 L tanks at 15°C with constant aeration, a 15:9 light:dark photoperiod, and a photon flux density of 55 µmol photons m^−2^ s^−2^. The second culture, *U. australis*, was originally collected in Zeeland (the Netherlands) and was maintained at the aquaculture facilities of the Royal Netherlands Institute for Sea Research (NIOZ; *n* = 2). The third culture, *U. lacinulata*, was originally collected in Texel (the Netherlands) and likewise cultivated at the aquaculture facilities at NIOZ, the Netherlands (*n* = 2). Both NIOZ strains had been in culture for 1 year at the moment of sampling. In addition, two individuals from a natural population in Zeeland, the Netherlands (51°38′35.3″N, 3°42′26.5″E) were collected. 1–2 cm^2^ tissue from two different individuals was sampled from each culture or site. All samples were rinsed in autoclaved seawater and immediately stored at −80°C.

All cultures and sampled individuals looked healthy except for the *U. australis* aquaculture samples. Most individuals in this culture looked healthy at first but suddenly sprouted white spots that continuously became larger until the entire tissue degraded. Sporulation was not observed in any of the tissues.

### RNA and DNA extraction and sequencing

Total DNA and RNA were extracted following the NetoVIR protocol optimized for viral metagenomics (30). Briefly, all samples were homogenized with 2.8 mm zirconium oxide beads, centrifuged, and filtered (using a 0.8 µm filter) to remove prokaryotic and eukaryotic organisms, as well as cellular debris. Subsequently, the samples were treated with benzonase (VWR) and micrococcal nuclease (NEB) to remove free-floating nucleic acids. DNA and RNA were extracted with the QiaAmp Viral RNA Mini kit (QIAGEN). Nucleic acids were randomly amplified with a modified whole-transcriptome amplification 2 (WTA2) kit procedure (Sigma-Aldrich). The amplified products were purified using the MSBSpin PCRapace purification kit (INVITEK), and the final sequencing library was prepared using the Nextera XT kit (Illumina). Sequencing of the samples was performed on the NextSeq500 platform (Illumina) for 300 cycles (2 × 150 bp paired ends), with an estimated 10 million reads per sample.

### Bioinformatics

Obtained raw reads were processed with ViPER (https://github.com/Matthijnssenslab/ViPER). Briefly, the raw Illumina reads were filtered for quality, and adapters were trimmed using Trimmomatic v0.39. This resulted in a total of 60,952,693 high-quality reads (*U. fenestrata* culture replicate 1 = 1,274,891 reads; *U. fenestrata* culture replicate 2 = 11,243,808; *U. australis* culture replicate 1 = 7,628,339; *U. australis* culture replicate 2 = 12,926,447; *U. lacinulata* culture replicate 1 = 4,071,840; *U. lacinulata* culture replicate 2 = 4,027,069; *U. australis* natural population replicate 1 = 9,995,302; *U. australis* natural population replicate 2 = 9,784,997). The reads were subsequently mapped with Bowtie2 v2.4.2 on the very sensitive setting to a reference *Ulva* genome (BioProject PRJEB25750) (66) to remove host-derived reads and to an assembled contaminome from a sequenced negative control to remove possible contamination. The remaining high-quality reads were *de novo* assembled into contigs using metaSPAdes v3.15. Contigs were then filtered on a length of at least 500 bp and clustered at 95% nucleotide identity over 85% of the length of the shortest contig to remove redundancy in the data using BLAST v2.11 (67) and the clustering algorithm shipped with the CheckV package (68). All reads were then mapped to the non-redundant contigs with bwa-mem2 v2.2.1 (69). The abundance of contigs that were less than 50% covered was set to 0 to exclude false positive detections. All contigs were classified by DIAMOND v2.0.11 on the sensitive setting against the NCBI nr database (70). Finally, KronaTools v2.8 (71) and the Phyloseq R package were used to visualize data (72, 73).

### Phylogenetic analyses

Contigs annotated as eukaryotic viruses were retained, and putative new viruses were identified (only contigs with >500 reads that likely represented near-complete genomes were considered). For each of the putatively new viruses, ORFs were predicted using ORFfinder (https://www.ncbi.nlm.nih.gov/orffinder/). Phylogenetic trees were generated based on amino acid sequences of the RNA-dependent RNA-polymerase for the RNA viruses (proteinase + RdRp polymerase in the case of the *Picornavirales*), and replicase for the CRESS DNA viruses. Reference amino acid sequences were retrieved from NCBI GenBank. The sequences were aligned with MAFFT v7.154b (74) and trimmed with trimAl v1.2rev59 (using gappyout settings) (75). Maximum likelihood phylogenetic trees were generated with IQ-TREE v1.6.12 on an automated model finder with at least 10,000 ultrafast bootstraps (76).

The genome of one putative new virus (see Results; Ulva picorna-like virus 1) was represented by two fragmented contigs. The largest contig was first extended using ContigExtender (77), which revealed the correct orientation of the second contig in relation to the viral genome. To obtain an accurate full genome for this virus, the missing overlapping fragment between the two contigs was sequenced with Sanger sequencing. The PCR was performed with the OneStep RT-PCR kit (Qiagen) using the following primers: TGGTTTGGTTGCTTTTCGGT (forward) and CAGCGTTAACAACCATGCGT (reverse). The thermal profile was set to: RT (50°C, 30 min), initial denaturation (95°C, 15 min), 40 cycles (denaturation at 94°C for 30 s, annealing at 51°C for 30 s, and extension at 72°C for 90 s), final extension (72°C, 10 min). Finally, the complete genome was assembled using Geneious Prime (v2022.1.1).

### qRT-PCR analyses

To verify the presence or absence of the putative new viruses in each of the samples, SYBR Green qRT-PCRs were performed with the *Power* SYBR Green RNA-to-CT *1-Step* Kit (Applied Biosystems) on an Applied Biosystems 7500 Real-Time PCR System. Primers for each virus were designed with Geneious Prime v2023.1.2 (see Table S1). Each reaction consisted of 2 µL sample, 10 µL *Power* SYBR mix, 1 µL of 10 µM forward primer, 1 µL of 10 µM reverse primer, and 0.16 µL RT enzyme mix, complemented with H_2_O to a total reaction volume of 20 µL. Thermal cycling conditions comprised a 48°C reverse transcription step for 30 min and a 95°C denaturation step for 10 min initially, followed by 40 cycles of 95°C for 15 s and 60°C for 1 min as per manufacturer’s instructions. All samples were quantified in duplicate for each virus, and average Ct (cycle threshold) values of the duplicates were calculated.

## Data Availability

The genome sequences of the putative new viruses are archived at GenBank (accession numbers OP924572–OP924576 and OP924591–OP924605), and raw sequencing data is archived at SRA (BioProject PRJNA902394).

## SUPPLEMENTAL MATERIAL

Fig. S1, Table S1 spectrum.00255-23-s0001.docx Figure S1: heatmap of 20 putative new viruses based on read number; Table S1: qPCR details.

ASM does not own the copyrights to Supplemental Material that may be linked to, or accessed through, an article. The authors have granted ASM a non-exclusive, world-wide license to publish the Supplemental Material files. Please contact the corresponding author directly for reuse.
