# Supplementary material for: Highly divergent CRESS DNA and picorna-like viruses associated with bleached thalli of the green seaweed Ulva
Source: Microbiol Spectr. 2023 Sep 19;11(5):e00255-23. doi: 10.1128/spectrum.00255-23 (PMC10581178; doi:10.1128/spectrum.00255-23)
Supplement: Fig. S1, Table S1 — Figure S1: heatmap of 20 putative new viruses based on read number; Table S1: qPCR details. [file spectrum.00255-23-s0001.docx]

Supplemental materials

**Highly divergent CRESS and picorna-like viruses associated with bleached thalli of the green seaweed *Ulva***

Luna M. van der Loos^a,e^, Lander De Coninck^b^, Roland Zell^c^, Sebastian Lequime^d^, Anne Willems^e^, Olivier De Clerck^a^, Jelle Matthijnssens^b^

^a^ Phycology Research Group, Department of Biology, Ghent University, Ghent, Belgium

^b^ KU Leuven, Department of Microbiology, Immunology and Transplantation, Rega Institute, Laboratory of Clinical and Epidemiological Virology, Laboratory of Viral Metagenomics, Leuven, Belgium

^c^ Section of Experimental Virology, Institute for Medical Microbiology, Jena University Hospital, Friedrich Schiller University, Jena, Germany

^d^ Cluster of Microbial Ecology, Groningen Institute for Evolutionary Life Sciences, University of Groningen, Groningen, The Netherlands

^e^ Laboratory of Microbiology, Department Biochemistry and Microbiology, Ghent University, Ghent, Belgium

Corresponding author: Luna M. van der Loos ([luna.vanderloos@ugent.be](mailto:luna.vanderloos@ugent.be))


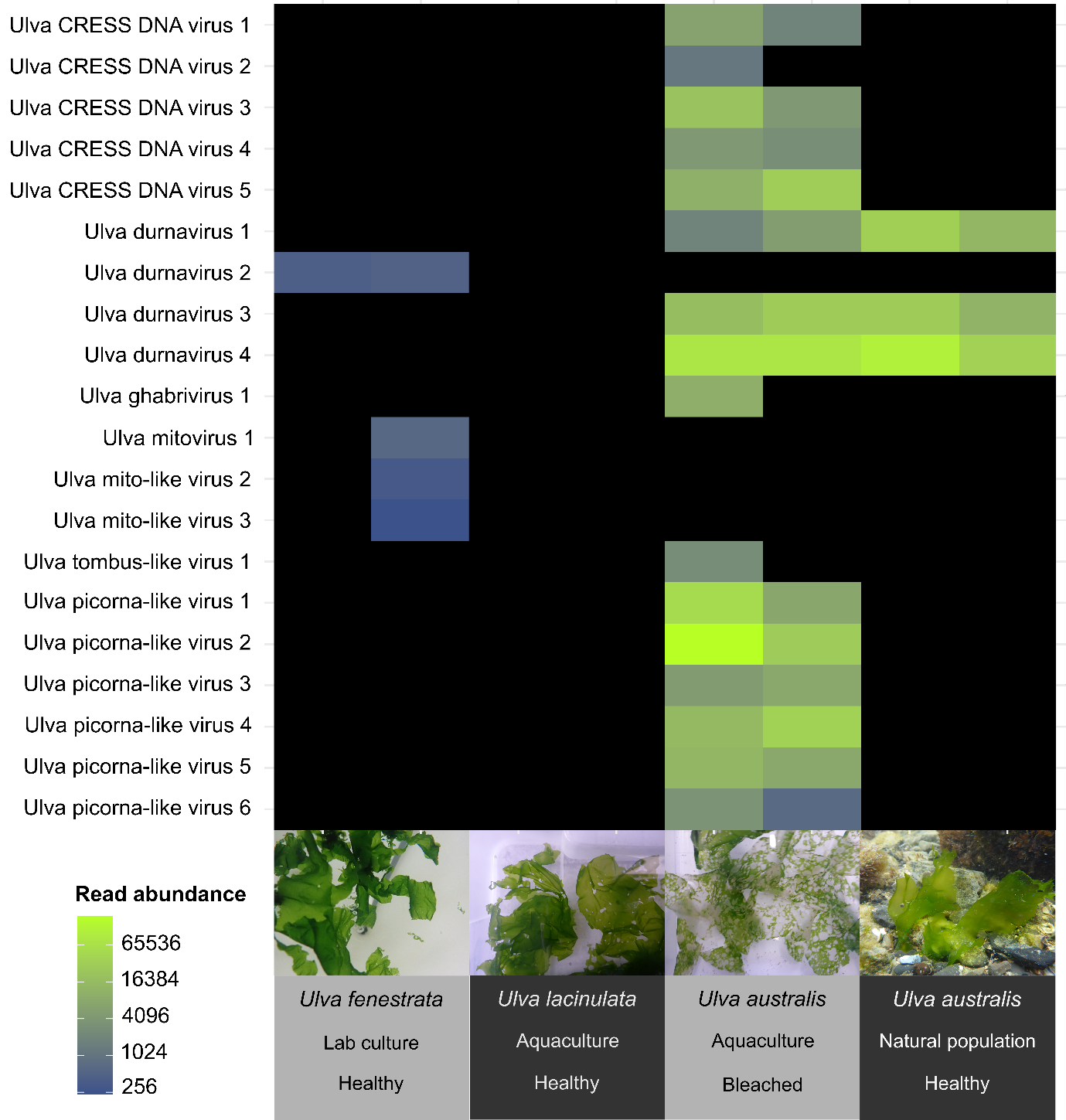


**Figure S1.** Heatmap showing normalized read number for the 20 putative new viruses associated with the green seaweed *Ulva*. From each culture or site, 1-2 cm^2^ tissue from two different individuals was sampled.

| **Name** | **Target** | **Minimum position** | **Maximum position** | **Direction** | **Sequence** | **Product Size** |
| --- | --- | --- | --- | --- | --- | --- |
| 1,885 F | Ulva CRESS virus 1 | 1885 | 1904 | forward | CAGATTTATGGGCACGCACG | 140 |
| 2,024 R | Ulva CRESS virus 1 | 2005 | 2024 | reverse | ATCCCTTCCCTCTCCCCTTC | 140 |
| 885 F | Ulva CRESS virus 2 | 885 | 904 | forward | ACTCGAAACACACGTCCTCC | 173 |
| 1,057 R | Ulva CRESS virus 2 | 1038 | 1057 | reverse | CGGTGTATGTGATCTCGCGA | 173 |
| 1,573 F | Ulva CRESS virus 3 | 1573 | 1592 | forward | GCAGTACAACCGTGTCCTGA | 255 |
| 1,827 R | Ulva CRESS virus 3 | 1808 | 1827 | reverse | ATGGTGGTATCCTGGAGGCT | 255 |
| 487 F | Ulva CRESS virus 4 | 487 | 506 | forward | ACTATGGTCGCTATGCAGGC | 144 |
| 630 R | Ulva CRESS virus 4 | 611 | 630 | reverse | TCCTCTCAGACTCTCCAGGC | 144 |
| 2,316 F | Ulva CRESS virus 5 | 2316 | 2335 | forward | CAACAAGGTGGGCAAGTTCG | 108 |
| 2,423 R | Ulva CRESS virus 5 | 2404 | 2423 | reverse | CCCTCGAAGTTCGTGAGCTT | 108 |
| 2,307 R | Ulva durnavirus 1 | 2287 | 2307 | reverse | GCATCATCTCCACAGACGACA | 136 |
| 2,172 F | Ulva durnavirus 1 | 2172 | 2191 | forward | GTGTTCCTTCAGGCCATCCA | 136 |
| 2,336 R | Ulva durnavirus 2 | 2316 | 2336 | reverse | AGACAAGAGCATCATCACCGT | 150 |
| 2,187 F | Ulva durnavirus 2 | 2187 | 2206 | forward | ATCGTGGAGTACCTTCGGGA | 150 |
| 96 F | Ulva durnavirus 3 | 96 | 115 | forward | ATGGGTACTTTGCCGGACTG | 172 |
| 267 R | Ulva durnavirus 3 | 248 | 267 | reverse | CACAGTTTCCTTCGAGGGGT | 172 |
| 1,305 F | Ulva durnavirus 4 | 1305 | 1324 | forward | AGACGCGGAGTTGGTATTCG | 291 |
| 1,595 R | Ulva durnavirus 4 | 1576 | 1595 | reverse | ATGTAGCTCACCTGCGCTTT | 291 |
| 637 F | Ulva ghabrivirus 1 | 637 | 656 | forward | TTGGGCTAGAGCAAAAGGGG | 174 |
| 810 R | Ulva ghabrivirus 1 | 791 | 810 | reverse | GCACGATACTGGCTGCAATG | 174 |
| 1,821 F | Ulva mito-like virus 2 | 1821 | 1840 | forward | TCAAGGGATGAGCTGCAGTG | 218 |
| 2,038 R | Ulva mito-like virus 2 | 2019 | 2038 | reverse | AAGCAGCGTTAACGGGATCA | 218 |
| 2,262 F | Ulva mito-like virus 3 | 2262 | 2281 | forward | CGACTCATTGATCCCCTCGG | 180 |
| 2,441 R | Ulva mito-like virus 3 | 2422 | 2441 | reverse | CCCCGACTCAGCGATTACTC | 180 |
| 1,527 F | Ulva mitovirus 1 | 1527 | 1546 | forward | TCCTTCTCTGATCTCCGCCA | 130 |
| 1,656 R | Ulva mitovirus 1 | 1637 | 1656 | reverse | TAGCTCCTCTGGTTGCCTCT | 130 |
| 351 F | Ulva picorna-like virus 1 | 351 | 370 | forward | TGTCTTTGTGCGCACGAAAG | 207 |
| 557 R | Ulva picorna-like virus 1 | 538 | 557 | reverse | GAAACTTTGCGGGGTTGTCC | 207 |
| 5,550 F | Ulva picorna-like virus 2 | 5550 | 5569 | forward | GACGACGCTGAGTACGAGAG | 200 |
| 5,749 R | Ulva picorna-like virus 2 | 5730 | 5749 | reverse | GCGATCTCAAGAGGGACGAG | 200 |
| 3,179 F | Ulva picorna-like virus 3 | 3179 | 3198 | forward | GTCACTCGATCCCCTGCAAA | 285 |
| 3,463 R | Ulva picorna-like virus 3 | 3444 | 3463 | reverse | CTCAGGTGTGAGTTCGCCTT | 285 |
| 7,106 F | Ulva picorna-like virus 4 | 7106 | 7125 | forward | ATGGCACCCTCAACTTACCG | 112 |
| 7,217 R | Ulva picorna-like virus 4 | 7198 | 7217 | reverse | GTTGGGGAGGTTAAAGCGGA | 112 |
| 1,112 F | Ulva picorna-like virus 5 | 1112 | 1131 | forward | TCTTGGCAAAAGAAACGCGG | 214 |
| 1,325 R | Ulva picorna-like virus 5 | 1306 | 1325 | reverse | TGGCTTCACGCATGGTTTTG | 214 |
| 2,928 F | Ulva picorna-like virus 6 | 2928 | 2947 | forward | CCTCAAGAAAGGGGGCTCAG | 290 |
| 3,217 R | Ulva picorna-like virus 6 | 3198 | 3217 | reverse | GTCTGGCGTACCCATCGTAG | 290 |
| 890 F | Ulva tombus-like virus 1 | 890 | 909 | forward | GACCACAGCCTAAACGTCGA | 175 |
| 1,064 R | Ulva tombus-like virus 1 | 1045 | 1064 | reverse | CGTCCGTCGTCGCATACTTA | 175 |

**Table S1.** Overview and details of the qPCR primers used in this study.
